# Inflammatory response of leptomeninges to a single cortical spreading depolarization

**DOI:** 10.1186/s10194-024-01823-1

**Published:** 2024-07-16

**Authors:** Anna A. Karan, Konstantin A. Gerasimov, Yulia S. Spivak, Elena M. Suleymanova, Lyudmila V. Vinogradova

**Affiliations:** 1 Department of Molecular Neurobiology, Institute of Higher Nervous Activity and Neurophysiology, Russian Academy of Sciences, Butlerova Street 5A, 117485 Moscow, Russia; 2 Pirogov Russian National Research Medical University, Ostrovityanova Street 1, 117997 Moscow, Russia

**Keywords:** Spreading depolarization, Meninges, Inflammation, Cytokines, Migraine aura, Headache, Interleukin-1beta, Tumor necrosis factor, CCL2, Fractalkine

## Abstract

**Background:**

Neurogenic meningeal inflammation is regarded as a key driver of migraine headache. Multiple evidence show importance of inflammatory processes in the dura mater for pain generation but contribution of the leptomeninges is less clear. We assessed effects of cortical spreading depolarization (CSD), the pathophysiological mechanism of migraine aura, on expression of inflammatory mediators in the leptomeninges.

**Methods:**

A single CSD event was produced by a focal unilateral microdamage of the cortex in freely behaving rats. Three hours later intact cortical leptomeninges and parenchyma of ipsi-lesional (invaded by CSD) and sham-treated contra-lesional (unaffected by CSD) hemispheres were collected and mRNA levels of genes associated with inflammation (*Il1b*, *Tnf*, *Ccl2; Cx3cl1*, *Zc3h12a*) and endocannabinoid CB2 receptors (*Cnr2*) were measured using qPCR.

**Results:**

Three hours after a single unilateral CSD, most inflammatory factors changed their expression levels in the leptomeninges, mainly on the side of CSD. The meninges overlying affected cortex increased mRNA expression of all proinflammatory cytokines (*Il1b*, *Tnf*, *Ccl2)* and anti-inflammatory factors *Zc3h12a* and *Cx3cl1*. Upregulation of proinflammatory cytokines was found in both meninges and parenchyma while anti-inflammatory markers increased only meningeal expression.

**Conclusion:**

A single CSD is sufficient to produce pronounced leptomeningeal inflammation that lasts for at least three hours and involves mostly meninges overlying the cortex affected by CSD. The prolonged post-CSD inflammation of the leptomeninges can contribute to mechanisms of headache generation following aura phase of migraine attack.

**Supplementary Information:**

The online version contains supplementary material available at 10.1186/s10194-024-01823-1.

## Background

Meningeal inflammation is regarded as a causal factor in primary and secondary headaches and a target for their therapy [1, 2]. According to the inflammatory hypothesis of migraine supported by numerous clinical findings, activation and sensitization of trigeminal afferents is mediated by local sterile inflammation of meninges [3]. Parameningeal inflammatory activity as well as increased serum levels of proinflammatory cytokines have been reported during and after migraine attacks in patients [4–6]. Despite the accepted meningeal origin of intracranial pain signals, the meninges remain to be among the most understudied tissues.

Cortical spreading depolarization (CSD), a self-propagating wave of transient local depolarization of cortical parenchyma, underlies aura symptoms preceding headache in a subpopulation of migraine patients. Data from animal models suggest that CSD can be an endogenous driver of meningeal inflammation and nociceptive signaling in migraine [7, 8]. It has been shown that CSD stimulates production of proinflammatory markers in the cerebral cortex [9–12] and activates dural trigeminovascular nociception [13]. CSD is supposed to trigger local sterile inflammation of the meninges either directly via diffusion of algesic factors from the cortical parenchyma to overlying meninges or indirectly via activation of resident meningeal immune cells and secretion of proinflammatory cytokines [7, 10, 14].

The cerebral meninges comprise the dura mater and leptomeninges (pia mater and arachnoid) both of which are richly vascularized and innervated. Most hypotheses have focused on the role of the dura mater and its nociceptors in generation of migraine pain. An important role dural neurogenic inflammation in migraine pathophysiology has been shown in many studies [7, 14]. It is thought that molecules released by cortical cells during CSD diffuse from the parenchyma to leptomeninges overlaying the affected cortical region and produce neurogenic inflammation and activation of dural afferents via an axon reflex [7, 14]. The dural inflammation and afferent activation have been shown to persist for one hour after CSD termination [10, 14] that may not be sufficient to explain the longer duration of the headache phase of migraine.

Contribution of the leptomeningeal pathophysiological processes to migraine headache is less clear although it has been suggested that long-term low-grade activation of pial nociceptors may be important for the initiation of headache phase of migraine [15]. It is generally thought that the cortical leptomeninges are pain-insensitive [16] but recent studies in awake patients have demonstrated that direct stimulation of the pia mater and small cerebral vessels produce pain [17]. Similarity of pain perceptions in migraine and subarachnoid hemorrhage also indicates an importance of pial nociception in headache generation. Failure of CGRP treatments in some migraine patients indicates a larger role of trigeminal leptomeningeal afferents in mediating migraine pain than previously thought [18].

Ability of CSD to activate programs of acute inflammatory gene expression in cortical parenchyma is well known [9–12] but there is no data about transcriptional inflammatory response of the meninges to CSD. Recent evidence shows that a single CSD activates meningeal immune cells [19] and its nociceptive afferents [20]. However, a very small population of dural afferents was activated in awake mice [20]. CSD-induced activation of macrophages in the pia mater started earlier and lasted longer than that in the dura mater [19] suggesting involvement of leptomeninges in general meningeal inflammation triggered by CSD. Recently, we showed that leptomeningeal expression of immune markers is altered by seizures (Karan et al., 2024) and hypothesized that CSD can also modify inflammatory status of the leptomeninges. Here, we examined whether a single CSD affects cytokine mRNA levels in the leptomeninges three hours later, i.e. at the time point corresponded to the timing of headache phase following aura. Unilateral CSD was induced in awake freely behaving rats and leptomeningeal mRNA expression of next immune-relevant molecules was assessed using quantitative real-time polymerase chain reaction (qPCR) analysis:

1. proinflammatory cytokines interleukin 1 beta (*Il1b)*, C–C motif chemokine ligand 2 (*Ccl2*) and tumor necrosis factor (*Tnf*)—major drivers of inflammatory responses. It has been previously shown that CSD upregulates the cytokines in the cortical parenchyma three-four hours after a single CSD [10–12] but it is unknown whether CSD affects expression of the proinflammatory markers in the leptomeninges.
2. anti-inflammatory factors C-X3-C Motif Chemokine Ligand 1 (*Cx3cl1*) and regnase-1/monocyte chemoattractant protein-induced protein 1/MCPIP1 (*Zc3h12a*). Fractalkine (CX3CL1) is known to control the severity and duration of neuroinflammation [21]. ZC3H12A is a negative regulator of inflammatory signaling pathways and degradation of inflammatory mRNAs such as *Il1b* [22]. Seizures change expression of the genes in both brain parenchyma and leptomeninges [23]. Involvement of the anti-inflammatory mediators in brain response to CSD has never been studied previously.
3. a modulator of neuro-immune interactions endocannabinoid CB2 receptor (*Cnr2*). It is thought that brain CB2 receptors are implicated in pain perception [24] and can restrain inflammatory processes and cytokines release during various brain insults [25]. Expression of cannabinoid type 2 receptors in the brain dramatically increases under inflammatory conditions [26]. Seizure-induced neuroinflammation is associated with pronounced changes in *Cnr2* expression in the leptomeninges and brain parenchyma [23]. We suggested that CSD may affect mRNA levels of CB2 receptors.

Also, parenchymal expression of *Ccl2*, *Cx3cl1*, *Zc3h12a, Cnr2* was evaluated three hours after CSD. Upregulation of proinflammatory cytokines *Il1b* and *Tnf* in the cortical parenchyma at the same time point after a single CSD has been shown in our previous study [12]. CSD-induced upregulation of *Ccl2* in the cerebral cortex has been reported in anesthetized mice [11]. Effects of CSD on expression of anti-inflammatory factors and CB2 receptors in the brain have never been assessed.

## Methods

### Animals

Twenty-nine adult male Wistar rats weighing 250–300 g (Scientific center for Biomedical Technologies of the Federal Medical and Biological Agency, Russia) were used. Animals were housed under the controlled environmental conditions (22˚C ± 2˚C, a 12-h light/dark cycle, lights on at 08.00 h) with food and water ad libitum. Animals were kept 5 per cage before surgery and individually after guide cannulas implantation. All experimental procedures were carried out according to the ARRIVE guidelines and the Directive 2010/63/EU for animal experiments. The study protocol was approved by the Ethics Committee of the Institute of Higher Nervous Activity and Neurophysiology of the Russian Academy of Sciences (protocol N1 from 01.02.2022). Every effort was made to minimize animal suffering and to ensure reliability of the results.

### Experimental groups

Animals were randomly assigned to two experimental groups using random.choice() command in the Python language: (1) CSD/sham group of rats with preliminary implanted bilateral guide cannulas for initiation of CSD and sham stimulation (*n* = 15). Since one animal was lost soon after the surgery, 14 rats of the group were subjected to PCR analysis. (2) Intact control group of naïve rats which did not undergo the surgery and cannula implantation (*n* = 14).

### Surgical intervention

Rats of CSD/sham group were bilaterally implanted with stainless-steel guide cannulas (23G) for CSD induction and sham treatment. Under anesthesia (400 mg/kg chloral hydrate, i.p., AppliChem, Darmstadt, Germany), guide cannulas were stereotaxically introduced in the homotopic areas of the primary somatosensory cortex (AP: − 2.8, ML: 4.8, and DV: 1.5) of the two hemispheres. The cannulas were fixed on the skull with acrylic dental plastic. A stylus of the same length as the guide cannula was inserted into it to prevent clogging. Experiments started two weeks after the surgery. All animals were pre-handled and habituated to the stylus removal daily a week before the start of experiments. Intact animals of the control group were subjected to the identical handling procedure.

### CSD induction and sham treatment in freely behaving rats

A single CSD was induced using the method of controlled microinjury of cortical parenchyma described in our previous studies [12, 27, 28]. Briefly, an awake rat was gently handled, and the injection cannula (0.3 mm diameter) was inserted via preliminary implanted guide cannula in the cortical parenchyma, extending 1.0 mm from the tip of the guide cannula. As shown previously, such cortical microinjury reliably produces a single unilateral CSD propagating over the whole ipsi-lesional cortex, including the frontal [12, 27] and retrosplenial (unpublished data) regions. The contralateral cortex underwent to the same cannula implantation was used as sham control. Sham stimulation was produced by insertion of a short injection cannula not extending from the guide cannula into cortical parenchyma.

### Experimental design

Freely behaving rats were individually placed in a transparent experimental chamber equipped with camera for video recording. After a 5-min habituation period, rats were subjected to a sham stimulation of the right cortex and 10 min later to a focal injury of the left cortex. Behavior of rats was video-recorded during 10 min after the sham- and CSD-inducing treatments. A single unilateral CSD was induced in each rat. Three hours after CSD induction, the animals were sacrificed by rapid decapitation and tissue samples from undamaged leptomeninges and cortical parenchyma located far from the implanted guide cannula were collected from the two hemispheres.

Rats of intact control group were individually placed in the same experimental chamber, their behavior was video-recorded for 30-min period and three hours later the animals were sacrificed. Samples of the control rats were gathered on the same day as rats with CSD induction.

We confined our study to analysis of mRNA levels that reflect well transcription factor activity, epigenetic regulation and RNA processing events.

### Sample isolation

The parenchymal and leptomeningeal mRNA levels of the inflammation-associated molecules and CB2 receptors were examined in the undamaged regions of the cortical meninges and parenchyma. Leptomeningeal samples included the arachnoid mater and pia mater from the intact (frontal, lateral, and ventral) surface of the left (CSD-affected) and right (sham-treated) cerebral hemispheres, separately. The meninges from the injury site were not included in the samples. The leptomeninges were identified based on extensive network of blood vessels and fibrous structure. Isolation of the leptomeninges was performed on a cooling plate at a temperature of -20C using ultrathin tweezers. The meninges were slowly separated from the cortical surface and occasional admixture of cortical tissue was carefully removed with tweezers or by pulling over filter paper. Samples of cortical parenchyma were collected from the frontal and retrosplenial regions of the left (CSD-affected) and right (sham-treated) cortices. The somatosensory cortex samples including the site of injury and the corresponding region of the sham hemisphere were gathered to control of the effect of injury on the expression of the studied genes. Tissue samples were collected in tubes, frozen in liquid nitrogen, and stored at − 80 °C for further analysis.

### RNA isolation and reverse transcription

Total RNA was isolated using ExtractRNA reagent (Evrogen, Moscow, Russia) according to the protocol provided by the manufacturer. To reduce RNA loss during extraction of RNA from the small amount of leptomeningeal tissue, we used 2ul of 0,25% linear polyacrylamide (Helicon) as a co-precipitant. Before the reverse transcription, residual DNA in samples was removed by treatment with DNase I (1U/μL) (Thermo Scientific, Waltham, MA, USA) according to the protocol specified by the manufacturer; then an equimolar mixture of random decaprimer (Evrogen, Russia) and Oligo(dT)15 primer (Evrogen, Russia) was used; the concentration of each primer in the reaction was 1 µM. The reverse transcription was performed using MMLV revertase (Evrogen, Moscow, Russia). Murine RNase Inhibitor (New England Biolabs, Ipswich, MA, USA) was used to prevent possible degradation of RNA. After reverse transcription, the reaction mixture was diluted eightfold with deionized water. cDNA samples were stored at − 20 ◦C.

### qPCR

We analyzed mRNA expression of next genes *Il1b*, *Tnf*, *Ccl2*, *Zc3h12a*, *Cx3cl1* and *Cnr2* (CB2) receptors in the leptomeninges and *Ccl2*, *Zc3h12a*, *Cx3cl1* and *Cnr2* in the cortical parenchyma. We also aimed at estimation CGRP gene (*Calca*) expression in the leptomeninges (post-CSD upregulation of the gene in the cortical parenchyma was shown in our previous study [12]). However, we found that the absolute level of *Calca* mRNA was very low in the meninges. Given small amount of meningeal tissue used for PCR analysis, we were not be able to measure *Calca* mRNA accurately and did not include the data in the current paper. Full original data for all studied genes (with outliers), including *Calca*, are shown in Supplementary Table 1.

Relative mRNA quantities of each gene were evaluated by real-time PCR (rtPCR) using a 5X qPCRmix-HS SYBR + LowROX reaction mixture (Evrogen, Russia), according to the protocol specified by the manufacturer, on a Biorad CFX 384 Real Time PCR. Relative quantities of mRNAs for genes expressed in the parenchyma were normalized to the geometric mean of the mRNA expression levels for the *Ywhaz*, *Osbp*, and *Hprt1* housekeeping genes. Relative quantities of mRNA in the meninges were normalized to the geometric mean of the mRNA expression levels for the *Ywhaz* and *Hprt1* genes. To assess the quality of the DNase treatment for all the samples and genes, we ran a negative control with the product of DNase I treatment. Gene expression was analyzed by the E − ΔΔCt method. The sequences of primers used are shown in Supplementary Table 2.

### Data analysis

The data are presented as mean ± standard deviation (SD). All statistical processing was carried out using programs written in the R language. An appropriate size of groups was calculated based on the data on *Il1b* expression from our previous study [12] using nomogram Altman with statistical power of 0.8. Since data distribution did not pass the normality Shapiro–Wilk test (shapiro.test() function), the significance of differences between CSD and intact control groups was evaluated using the Mann–Whitney test (wilcox.test(paired = FALSE) function, the level of significance was *p* < 0.05. All groups were examined for the presence of outliers using the 1.5xIQR rule; the outlying values were automatically excluded from analysis by software. The significance of differences between the affected and sham-treated hemispheres in CSD group was evaluated using the Wilcoxon test (wilcox.test(paired = TRUE) function, the level of significance was *p* < 0.05 without exclusion of the outlying values. Investigator (A.K.) who performed statistical analysis was blinded to the group assignment. Full original data for all studied genes (with outliers) and results of statistical analysis are shown in Supplementary Tables 1 and 3.

## Results

### Expression of genes associated with inflammation in the leptomeninges

#### Effects of a single CSD on leptomeningeal expression of inflammatory markers (CSD vs sham control)

**Fig. 1:**
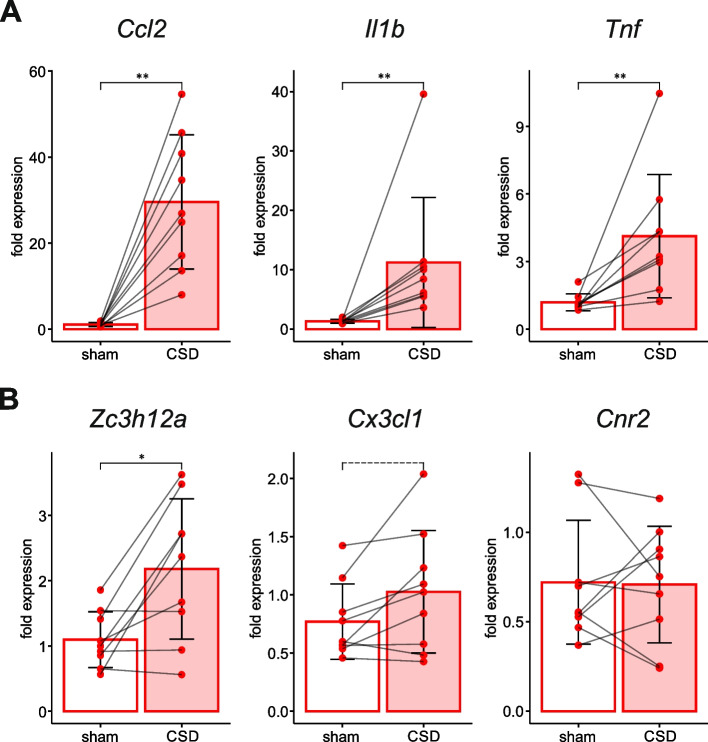
Effect of a single unilateral CSD on leptomeningeal expression of genes associated with inflammation (CSD vs sham control). Comparison of mRNA levels of pro-inflammatory cytokines *Ccl2*, *Il1b*, *Tnf* (**A**) and anti-inflammatory factors *Zc3h12a, Cx3cl1*, *Cnr2* (**B**) between the meninges overlying sham-treated contralateral cortex (sham) and affected by CSD cortex (CSD) at 3 h after induction of a single unilateral CSD (*n* = 9). Statistical outcome is shown: *—*p* < 0.05, **- *p* < 0.005. Dashed line marks a tendency to significant difference (0.05 < *p* < 0.1, see the text)

To assess effects of CSD on leptomeningeal inflammatory status, we compared mRNA levels of inflammatory marker in the leptomeninges overlying the cortex affected by CSD and contralateral sham-treated cortex unaffected by CSD in rats of CSD/sham group. Three hours after initiation of a single CSD, the leptomeninges of the two hemispheres differed in mRNA levels of all studied genes except *Cnr2* (Fig. 1). Expression of proinflammatory cytokines *Ccl2*, *Il1b* and *Tnf* and anti-inflammatory mediator *Zc3h12a* was significantly higher in the leptomeninges overlying the cortex affected by CSD than in the contralateral sham-treated meninges (Fig. 1). Levels of *Cx3cl1* mRNA also tended to be higher in the ipsilateral meninges than in the contralateral ones (*p* = 0.0547, Fig. 1B). Thus, a single unilateral CSD was followed by upregulation of the pro- and anti-inflammatory markers in the leptomeninges for at least three hours.

#### Effects of intracortical cannula implantation on leptomeningeal expression of inflammatory markers (sham vs intact control)

**Fig. 2:**
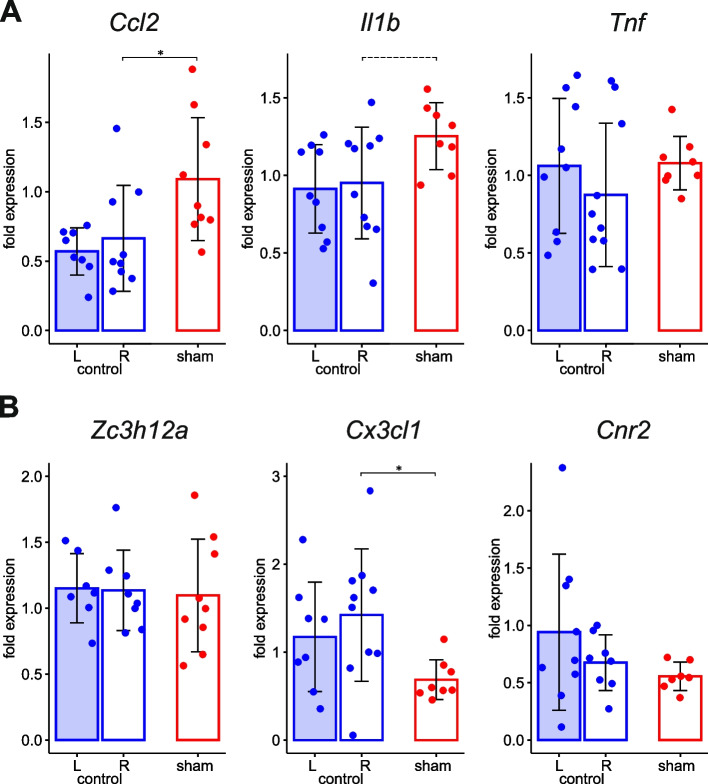
Effect of cannula implantation on leptomeningeal expression of genes associated with inflammation (sham vs intact control). mRNA levels of pro-inflammatory cytokines *Ccl2*, *Il1b*, *Tnf* (**A**) and anti-inflammatory factors *Zc3h12a, Cx3cl1*, *Cnr2* (**B**) in the meninges of the sham-treated hemisphere implanted with cannulas with respective levels in the right (R) and left (L) meninges of intact control rats at 3-h time point are compared. Statistical outcome of the between-group comparison is marked on each fragment by *—*p* < 0.05.Tendency (0.05 < *p* < 0.1) is shown by dashed line

To control potential contribution of sham surgery and implantation procedure to the meningeal inflammatory response, we compared expression of the genes in the leptomeninges of sham-treated hemisphere implanted with guide cannula and in undamaged leptomeninges of naïve rats from intact control group (Fig. 2). In naive animals, mean mRNA levels in the meninges of the two hemispheres did not differ significantly. Compared to intact controls, the meninges covering sham-treated cortex with implanted guide cannula showed slight upregulation of *Ccl2*, tendency to upregulation of *Il1b* (*p* = 0.0831) and significant downregulation of anti-inflammatory cytokine *Cx3cl1*(Fig. 2). That is, chronic implantation of intracortical cannulas produced a low-grade inflammatory process in the leptomeninges that persisted for at least two weeks after implantation surgery.

### Expression of genes associated with inflammation in the cortical parenchyma

#### Effects of a single CSD on parenchymal expression of inflammatory markers (CSD vs sham control)

**Fig. 3:**
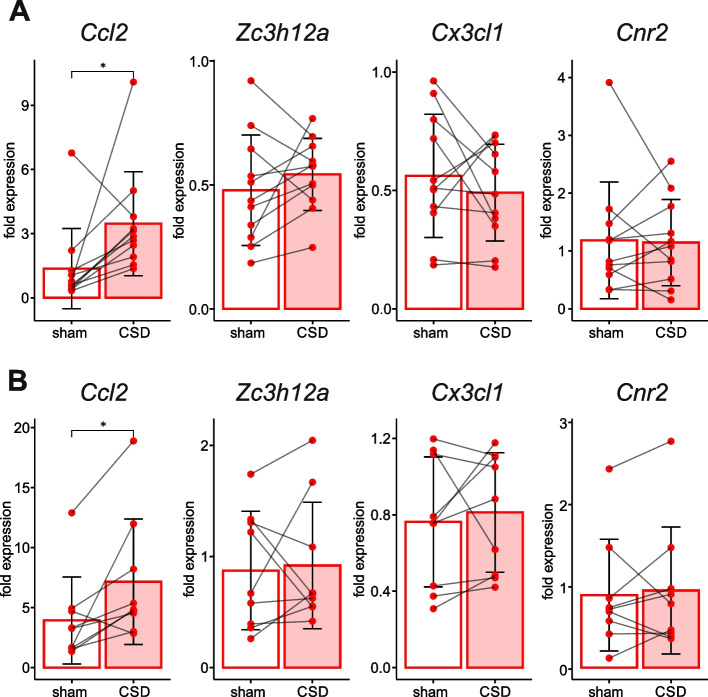
Effect of a single unilateral CSD on parenchymal expression of genes associated with inflammation (CSD vs sham control). mRNA levels of *Ccl2*, *Zc3h12a, Cx3cl1*, *Cnr2* in the parenchyma of the frontal (**A**) and retrosplenial (**B**) cortices of the sham-treated contralateral (sham) and affected by CSD ipsilateral (CSD) hemispheres at 3 h after induction of a single unilateral CSD (*n* = 9). Significant difference is marked by *—*p* < 0.05

In the same rats, parenchymal expression of the *Ccl2*, *Cx3cl1*, *Zc3h12a* and *Cnr2* was assessed three hours after a single CSD (Fig. 3). Post-CSD upregulation of IL1β and TNF mRNA in the ipsilateral cortical parenchyma at the same time point has been shown in our previous study (Volobueva et al., 2023). The parenchyma invaded by CSD exhibited higher expression of proinflammatory cytokine *Ccl2* than the contralateral unaffected cortex did (Fig. 3). Expression of anti-inflammatory factors in the cortical parenchyma did not differ from the sham-treated cortex at three hours after a single CSD (Fig 3).

#### Effects of intracortical cannula implantation on parenchymal expression of inflammatory markers (sham vs intact control)

**Fig. 4:**
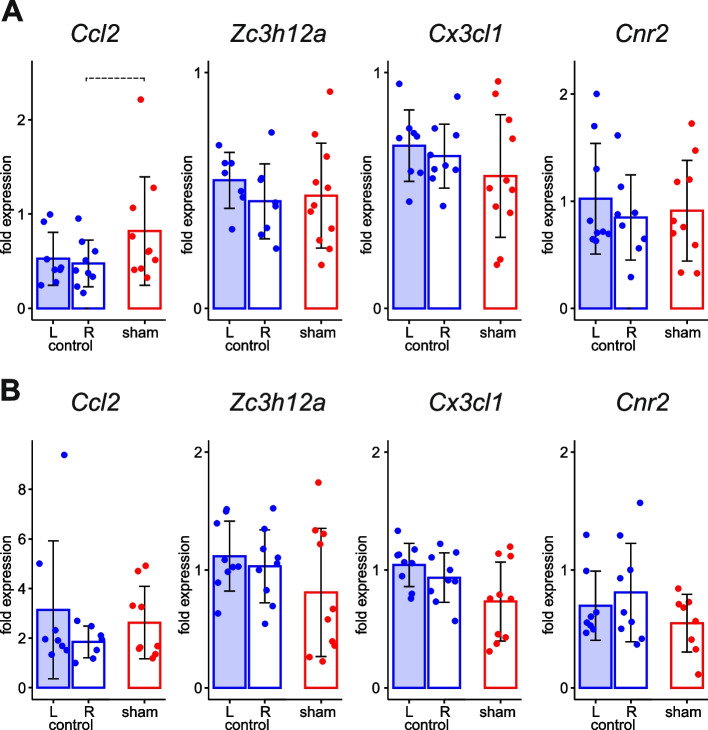
Effect of cannula implantation on parenchymal expression of genes associated with inflammation. Comparison of mRNA levels of *Ccl2*, *Zc3h12a, Cx3cl1* and *Cnr2* in the parenchyma of the frontal (**A**) and retrosplenial (**B**) cortices of the right sham-treated (R) cortex in CSD group with levels in the right (R) and left (L) cortical parenchyma of intact rats of control group at 3-h time point. In sham-treated cortex, guide cannula was implanted in the somatosensory cortex of the right hemisphere. Dashed lines mark tendencies to significant difference (0.05 < *p* < 0.1, see the text)

To estimate potential effects of intracortical cannula implantation on inflammatory status of cortical parenchyma, we compared mRNA levels of the studied genes in two cortical regions located far from the site of cannula implantation in sham and naïve controls. Only a subtle tendency to increased *Ccl2* expression (*p* = 0.0947) was found in the parenchyma implanted with a guide cannula (Fig. 4).

## Discussion

Our study shows for the first time that a single CSD produces significant inflammatory response of the overlying leptomeninges that persists for at least three hours after CSD termination. Given the timing of the aura phase relative to headache during migraine attack, the result provides additional support for a potential involvement of CSD in meningeal inflammatory mechanisms of pain generation in migraine with aura.

The leptomeninges and their vessels receive sensory fibers from the trigeminal ganglion [29] and recent data from awake patients have shown that the pia mater and small cerebral vessels are pain-sensitive [17]. Preclinical studies have shown that a single CSD produces rapid activation of pial macrophages [19] and most meningeal (pial) afferents [20]. It has been suggested that pial nociceptors are relevant to migraine with aura, whereas dural and extracranial nociceptors might be more important in migraine without aura [15].

Our study shows that leptomeninges of the affected hemisphere exhibit strong upregulation of proinflammatory cytokine genes *Il1b*, *Tnf* and *Ccl2* three hours after a single CSD. Proinflammatory response of cortical parenchyma to CSD has been intensively studied and increased production of the proinflammatory markers was found in the cerebral cortex within one-four hours after CSD induced by KCl, mechanical or optogenetic stimulation [10–12]. Here, we show that CSD upregulates the cytokine mRNA expression not only in the affected cortical parenchyma but also in the overlying leptomeninges. Increased expression of proinflammatory cytokines in the leptomeninges and brain parenchyma has been also found after seizures [23].

The result suggests that meningeal immune cells are involved in the lasting production of proinflammatory cytokines following CSD. The meninges are known to contain resident immune cells [30] and a single CSD rapidly activates meningeal macrophages [19]. Leptomeningeal mRNA levels of immune markers are not affected by non-resident cells although in the cortical parenchyma and dura mater blood cells contribute to the total mRNA pool of inflammatory mediators [31]. Activated pial macrophages can release pro- and anti-inflammatory factors into the subarachnoid space contributing to induction and resolution of inflammation, respectively.

Pro-inflammatory cytokines produced by meningeal immune cells may act as pain mediators promoting headache. Perfusion of the subarachnoid space with inflammatory factors activates second-order neurons in the trigeminal nucleus caudalis [32]. Meningeal application of IL1β and TNF sensitizes trigeminal afferents [33, 34]. Increased blood levels of IL1β and TNF have been reported in migraine patients [35]. The proinflammatory cytokine CCL2 is implicated in migraine pathophysiology [36] and exhibits high sensitivity to neuronal excitation [23]. Rapid upregulation of *Ccl2* mRNA after a single CSD has been shown in the cortical parenchyma of anesthetized mice [11] as well as in the dura mater and trigeminal ganglion tissues in the nitroglycerin model of migraine [36].

Meningeal macrophage activation is known to include anti-inflammatory response [19] and our study shows that CSD increases leptomeningeal expression of anti-inflammatory markers *Cx3cl1* and *Zc3h12a*. The molecules govern resolution of acute inflammation and limit its duration [21, 22]. Experimental evidence indicates a role of CX3CL1 signaling in pathogenesis of nociception and allodynia during neuropathic pain states [37]. Recently, increased levels of fractalkine (CX3CL1) were found in cerebrospinal fluid of migraine patients and suggested as a potential clinical biomarker of migraine [38]. In our study, a single CSD upregulated *Cx3cl1* in the meninges not changing its expression in the affected cortical parenchyma.

Cannula implantation produced long-lasting inflammatory process in the leptomeninges with upregulation of proinflammatory cytokines *Ccl2* and *Il1b* and downregulation of anti-inflammatory mediator *Cx3cl1*. The parenchyma exhibited milder transcriptional response to cannula implantation (only CCL2 tended to increase). Remarkably, the inflammatory response was detected in intact regions of the meninges and parenchyma distant from the site of cannula implantation. To our knowledge, this is the first study showing that implantation procedure with focal violation of meningeal integrity triggers their low-grade inflammation that persists for at least two weeks after the invasive implantation procedures. Reduction of CX3CL1 protein levels for two weeks after cortical injury has been reported in mice [39]. In the cortical parenchyma, fractalkine (CX3CL1) is known to play the key role in neuron-microglia communication [40]. Cortical neurons release fractalkine that acts on its cognate microglial receptors CX3CR1. Resident meningeal macrophages also express fractalkine receptor CX3CR1 [21, 30] and leptomeninges exhibit constitutive expression of CX3CL1 [31]. Given that fractalkine inhibits microglial activation and suppresses production of proinflammatory cytokines in the healthy CNS [40], meningeal downregulation of *Cx3cl1* may contribute to chronic meningeal inflammation triggered by cannula implantation.

Expression of cannabinoid type 2 receptors (*Cnr2*) is low in the healthy CNS but dramatically increases under inflammatory conditions [26]. It is believed that upregulation of brain CB2 receptors restrains inflammatory processes and cytokines release [25]. No changes in parenchymal and leptomeningeal mRNA levels of CB2 receptor was found in the present study. Previously, we have shown that brief epileptic seizures do not affect cortical levels of CB2 expression [41] but prolonged seizures produce delayed CB2 upregulation in both cortical parenchyma and leptomeninges [23]. It is possible that three-hour time point is too early for detection the slowly developing changes in CB2 receptor expression. Another possibility is that a single CSD represents too mild stimulus insufficient to change expression of CB2 receptors.

Unexpected finding of the present study is strikingly asymmetric meningeal response to unilateral CSD. The transcriptional cytokine response mainly involved the leptomeninges overlying the cortical tissue which had been affected by CSD three hours ago. The mainly unilateral inflammation of the leptomeninges may contribute to ipsilateral activation of the trigeminal ganglion and the trigeminal nucleus caudalis shown after CSD in rats [42] and be involved in unilateral nociceptive perception in humans. The post-aura headache usually develops on the affected side and painful events elicited by mechanical stimulation of the pia mater in patients were also referred ipsilaterally to the stimulus [17]. Given more restricted areal of CSD propagation in the gyrenchephalic cortex than that in the lissencephalic cortex of rodents [43], rather local leptomeningeal inflammatory response may be expected in migraine patients. Recent imaging data demonstrated inflammation in parameningeal tissue overlying the occipital cortex in migraine with aura patients [5].

The study has limitations. First, only male rats were studied. Given that migraine is more common in women than in men, patterns of meningeal inflammations in females need to be further investigated. Second, the study included only a single time point. It cannot be excluded that dynamics of changes in cytokine expression following CSD may differ in the meninges and parenchyma. To assess temporal profiles of parenchymal and meningeal responses to CSD, multiple time points should be studied.

## Conclusion

CSD is regarded as the neuronal correlate of migraine aura but its role in headache generation is still debated. The current study shows that a single unilateral CSD is sufficient to produce pronounced leptomeningeal inflammation persisting long after termination of the brief parenchymal event. The increased mRNA levels of pro-inflammatory cytokines were observed mainly in the leptomeninges overlying the affected cortex. Given that meningeal inflammatory mechanisms play an important role in migraine headache [1–3, 7] and inflammatory mediators can provide sustained stimulus driving unilateral activation/sensitization of trigeminal afferents, the neurogenic inflammation of meninges ipsilateral to CSD may contribute to pathogenic mechanisms of unilateral post-aura headache in migraine patients.

### Supplementary Information

Supplementary Material 1.

Supplementary material 2.

Supplementary Material 3.

## Data Availability

Data is provided as supplementary information files.

## Abbreviations

CB2 (*Cnr2*): Cannabinoid receptor type 2
CCL2: C-C Motif chemokine ligand 2
CSD: Cortical spreading depolarization
CX3CL1: C-X3-C motif chemokine ligand 1, fractalkine
IL1b: Interleukin 1β
qPCR: Quantitative real-time polymerase chain reaction
TNF: Tumor necrosis factor
ZC3H12A: Zinc finger CCCH-type containing 12A
