# Supplementary material for: Inflammatory response of leptomeninges to a single cortical spreading depolarization
Source: J Headache Pain. 2024 Jul 16;25(1):113. doi: 10.1186/s10194-024-01823-1 (PMC11251126; doi:10.1186/s10194-024-01823-1)
Supplement: Supplementary file 1 — Supplementary Material 1. [file 10194_2024_1823_MOESM1_ESM.pdf]

Supplementary Table S1. Raw data for mRNA levels of Ccl2, Il1b, Tnf, Cx3cl1, Zc3h12a, Cnr2 in the cortical meninges (CM), frontal cortex (FC), retrosplenial cortex (RC) and somatosensory cortex (SSC) in all rats of CSD and control groups.

CM

Sample	Group	*Ccl2*	*Il1b*	*Tnf*	*Zc3h12a*	*Cx3cl1*	*Cnr2*	*Calca*
1CMR	CSD	0.56568	~~2.00238~~	1.11717	0.85446	0.85263	0.52738	0.99884
2CMR	CSD	0.80964	1.43752	1.42481	0.99716	0.56823	0.72109	-
3CMR	CSD	1.12038	1.18499	0.97011	1.54046	0.56506	0.55438	-
4CMR	CSD	0.89927	1.00000	1.00000	0.91712	0.59865	0.46836	1.00000
5CMR	CSD	1.88580	1.56271	0.99696	0.56368	0.53712	~~1.32529~~	1.91368
6CMR	CSD	-	-	-	-	-	-	-
7CMR	CSD	1.34185	1.38114	0.84993	0.64907	0.45773	0.36947	-
8CMR	CSD	0.79616	0.93274	1.08748	1.07613	~~1.42346~~	0.54672	-
9CMR	CSD	1.67421	1.71012	1.44620	1.57815	0.99950	1.26542	-
10CMR	CSD	-	-	-	-	-	-	-
11CMR	CSD	0.76919	1.32459	1.18437	1.41121	0.77681	0.70041	-
15CMR	CSD	1.62700	1.20200	~~2.09890~~	1.85613	1.14561	~~1.27802~~	-
1kCMR	control	1.00000	1.17257	1.33256	1.00000	1.00000	1.00000	-
2kCMR	control	0.42592	0.65251	0.86949	1.03610	1.61849	0.68600	2.57014
3kCMR	control	0.28286	0.67089	0.75121	1.10766	1.70644	0.71653	3.50743
4kCMR	control	1.45229	1.47018	1.61017	~~2.80439~~	2.83641	~~1.72834~~	2.49321
5kCMR	control	~~2.42130~~	1.23864	1.56850	1.76078	1.87214	~~1.70401~~	-
6kCMR	control	0.54289	0.87696	0.66108	1.28729	1.81250	0.95704	1.13902
101CMR	control	0.37563	0.30522	0.58789	~~0.05980~~	0.05347	0.27114	-
102CMR	control	0.48135	0.72859	0.57814	0.81046	1.50940	0.52423	0.38527
103CMR	control	0.49289	1.20424	0.39551	0.83680	0.81916	0.49039	-
104CMR	control	0.92574	1.18992	0.39319	1.24609	0.98503	0.75530	-
1CML	CSD	17.10309	10.52619	3.08473	2.36678	1.09269	0.90603	3.39638
2CML	CSD	40.84350	8.44220	~~10.46186~~	2.71699	1.23280	0.65613	0.93841
3CML	CSD	24.89784	10.04801	4.33363	1.52481	0.57666	0.25118	2.50261
4CML	CSD	7.99432	3.64442	1.75302	0.93766	0.48203	0.24127	3.53894
5CML	CSD	54.61968	~~39.59090~~	5.75085	2.71787	0.84035	0.75291	5.44522
6CML	CSD	54.82415	13.47965	7.66235	~~10.18123~~	2.03253	0.85542	1.15657
7CML	CSD	34.66602	6.14862	1.23314	0.56036	0.42622	0.51482	-
8CML	CSD	13.59736	5.52847	3.22642	1.67038	1.52295	1.00328	-
9CML	CSD	0.05151	-	-	0.22246	6.17057	0.08757	-
10CML	CSD	27.75597	~~30.83012~~	3.66888	5.10274	1.62005	1.09044	3.62067
11CML	CSD	45.66128	11.37279	2.96782	3.47728	1.02445	0.86465	1.47154
15CML	CSD	26.89784	5.69466	4.33258	3.62288	2.03889	1.18986	-
1kCML	control	0.52694	0.86802	1.44270	1.43811	1.62253	1.40119	-
2kCML	control	~~1.17101~~	0.66427	1.16915	~~0.12138~~	0.35540	0.11308	-

	we delete in all experiments (№9)
	we delete in pair comparison
~~0.00000~~	outlier in comparison with control

3kCML	control	0.50858	0.52742	1.05006	1.51046	1.37454	0.94676	-
4kCML	control	0.75685	1.15048	1.56428	~~2.70057~~	~~3.39161~~	2.37196	3.40360
5kCML	control	0.64879	1.26109	1.64587	1.11336	2.28082	1.34980	1.61460
6kCML	control	0.24186	0.82727	0.48456	1.08454	1.38209	0.63161	-
101CML	control	0.70237	0.57062	0.57341	1.00683	0.94063	0.57630	1.29836
102CML	control	0.71062	1.15086	0.98928	1.16811	0.88693	0.69366	0.69973
103CML	control	0.46107	1.19411	0.63396	0.73358	0.54773	0.38662	-
104CML	control	-	-	-	-	-	-	-

FC

**Sample**	**Group**	***Ccl2***	***Zc3h12a***	***Cx3cl1***	***Cnr2***
1FCR	CSD	0.51506	0.53539	0.79986	1.17993
2FCR	CSD	1.27715	0.91990	0.96289	1.72490
3FCR	CSD	0.40609	0.18445	0.18501	0.32786
4FCR	CSD	0.32876	0.33760	0.50277	0.75893
5FCR	CSD	~~6.77421~~	0.73923	0.50955	~~3.91449~~
6FCR	CSD	0.60901	0.43564	0.54266	0.59092
7FCR	CSD	2.21282	0.28807	0.40537	1.47284
8FCR	CSD	0.76438	0.41109	0.91024	0.70040
9FCR	CSD	229.85598	8.98292	0.23052	3.96354
10FCR	CSD	0.42007	0.64488	0.71969	0.81655
11FCR	CSD	0.59983	0.25166	0.20771	0.33463
15FCR	CSD	1.06274	0.51057	0.43186	1.20195
1kFCR	control	0.16373	~~0.92009~~	0.73421	~~2.78304~~
2kFCR	control	0.40215	0.34111	0.59044	0.65105
3kFCR	control	0.37382	0.25351	0.43414	0.56018
4kFCR	control	-	-	-	-
5kFCR	control	0.95256	0.54560	0.55117	1.13633
6kFCR	control	0.70664	0.55260	0.58767	0.28961
101FCR	control	0.23031	0.43361	0.76380	0.89421
102FCR	control	0.50910	0.31243	0.60589	0.76930
103FCR	control	0.60525	0.74647	0.89837	1.61144
104FCR	control	0.33528	0.44869	0.65093	0.87626
1FCL	CSD	3.15234	0.57440	0.57980	1.77320
2FCL	CSD	2.68557	0.69448	0.65315	0.84841
3FCL	CSD	2.45478	0.24745	0.20276	0.51417
4FCL	CSD	1.36296	0.49862	0.73454	0.81840
5FCL	CSD	3.78974	0.59537	0.48461	2.08555
6FCL	CSD	1.54026	0.57872	0.70159	1.16712
7FCL	CSD	5.01199	0.76749	0.73277	2.55200
8FCL	CSD	~~10.08962~~	0.50507	0.38113	0.15525

9FCL	CSD	2.45454	0.61073	0.85770	0.45797
10FCL	CSD	3.23263	0.43857	0.34925	1.08153
11FCL	CSD	2.88074	0.40522	0.17571	0.30667
15FCL	CSD	1.90939	0.65604	0.40522	1.30676
1kFCL	control	0.99590	0.69230	0.57523	2.00091
2kFCL	control	0.24494	0.61568	0.72971	1.30234
3kFCL	control	0.40864	0.33553	~~0.30717~~	0.63012
4kFCL	control	-	-	-	-
5kFCL	control	~~3.54329~~	0.47085	0.45300	1.70258
6kFCL	control	0.91886	~~0.87001~~	0.95261	0.69594
101FCL	control	0.27756	~~0.24084~~	0.58142	0.64355
102FCL	control	0.43241	0.57424	0.72219	0.71397
103FCL	control	0.52138	0.61638	0.76341	0.70327
104FCL	control	0.40936	0.49716	0.74354	0.81930

RC

Sample	Group	*Ccl2*	*Zc3h12a*	*Cx3cl1*	*Cnr2*
1RCR	CSD	3.26023	0.66859	0.75518	0.72993
2RCR	CSD	-	-	-	-
3RCR	CSD	3.31864	0.26034	0.42764	0.56666
4RCR	CSD	1.62602	1.21953	1.13818	0.68096
5RCR	CSD	4.92768	1.74160	0.79012	~~2.41591~~
6RCR	CSD	4.69726	1.30590	1.11740	~~1.46106~~
7RCR	CSD	1.68961	0.58023	1.19691	0.84221
8RCR	CSD	1.18802	0.22386	0.45460	0.32944
9RCR	CSD	5.04610	0.44665	0.65294	0.92583
10RCR	CSD	1.58958	0.35789	0.30816	0.11494
11RCR	CSD	1.34703	0.39354	0.37391	0.40824
15RCR	CSD	~~12.89718~~	1.33618	0.75638	0.70923
1kRCR	control	~~5.62654~~	0.82840	0.72952	1.29253
2kRCR	control	1.00000	1.00000	1.00000	1.00000
3kRCR	control	1.94223	0.54249	0.56662	0.41553
4kRCR	control	-	-	-	-
5kRCR	control	~~8.59624~~	1.52134	0.82055	1.56914
6kRCR	control	2.69601	1.34934	0.91490	0.92936
101RCR	control	1.17823	0.69185	1.22127	0.50094
102RCR	control	2.48215	1.05607	1.10344	0.55873
103RCR	control	2.10601	1.17977	0.90547	0.36781
104RCR	control	1.51870	1.10540	1.14628	0.63957
1RCL	CSD	4.53011	~~1.66930~~	1.17675	0.96059
2RCL	CSD	8.67644	0.65976	0.58262	0.66215

3RCL	CSD	5.36379	0.67184	0.46988	0.35249
4RCL	CSD	4.73911	0.54615	0.61794	0.37022
5RCL	CSD	8.21787	~~2.04631~~	1.10120	~~2.75205~~
6RCL	CSD	2.83881	1.08566	1.04956	0.77616
7RCL	CSD	11.98232	0.62319	1.10866	1.45946
8RCL	CSD	-	-	-	-
9RCL	CSD	9.72814	0.92050	0.80608	0.30120
10RCL	CSD	3.00209	0.55252	0.48265	0.45799
11RCL	CSD	4.83205	0.41745	0.42065	0.41889
15RCL	CSD	~~18.89287~~	0.65994	0.88231	0.89163
1kRCL	control	1.95612	1.51562	0.94627	1.29766
2kRCL	control	9.38493	0.89133	0.79845	0.52803
3kRCL	control	1.51477	1.02221	0.75445	0.64014
4kRCL	control	-	-	-	-
5kRCL	control	~~16.44686~~	1.49772	1.13291	~~2.17531~~
6kRCL	control	1.90658	1.02882	1.05585	0.46655
101RCL	control	1.33891	0.63203	1.06450	0.55300
102RCL	control	5.00520	1.39605	1.33234	0.99156
103RCL	control	2.31960	0.98332	1.12456	0.60337
104RCL	control	1.68608	1.08338	1.17337	0.49578

SSC

Sample	Group	*Ccl2*	*Zc3h12a*	*Cx3cl1*	*Cnr2*
1SSCR	CSD	0.30408	0.75418	0.84673	1.72970
2SSCR	CSD	3.08379	1.11008	0.87583	2.42750
3SSCR	CSD	0.24187	0.10072	0.18785	0.55601
4SSCR	CSD	1.10242	0.80104	0.74034	2.31834
5SSCR	CSD	~~6.54203~~	1.06697	0.68254	~~7.20769~~
6SSCR	CSD	0.52403	0.67233	0.61560	1.80021
7SSCR	CSD	1.35915	0.38765	0.46239	1.04133
8SSCR	CSD	0.15701	0.51008	0.41030	0.54848
9SSCR	CSD	0.55513	0.16782	0.36538	0.34323
10SSCR	CSD	0.43158	0.65635	0.48348	0.71242
11SSCR	CSD	0.85988	0.22524	0.22358	0.30641
15SSCR	CSD	3.31843	0.65791	0.44570	0.87958
1kSSCR	control	0.62418	0.50915	0.48218	1.23476
2kSSCR	control	1.00000	~~1.00000~~	~~1.00000~~	1.00000
3kSSCR	control	0.59538	0.49986	0.54883	0.34615
4kSSCR	control	-	-	-	-
5kSSCR	control	~~7.28340~~	0.57509	0.58414	~~3.44326~~
6kSSCR	control	0.70671	0.42788	0.68157	0.38208

101SSCR	control	0.20396	0.40834	0.68589	0.58871
102SSCR	control	0.36258	0.45943	0.66250	1.00575
103SSCR	control	0.81870	0.68319	0.89475	0.91893
104SSCR	control	0.90465	0.54582	0.65361	0.80308
1SSCL	CSD	41.20002	1.16773	0.69285	0.51225
2SSCL	CSD	181.98181	3.66480	0.79295	1.10184
3SSCL	CSD	63.04836	0.69759	0.25707	0.41773
4SSCL	CSD	35.11102	1.45301	0.81082	0.62244
5SSCL	CSD	84.39240	4.51705	0.69062	4.93987
6SSCL	CSD	61.02389	1.93212	0.65085	0.82787
7SSCL	CSD	85.66478	2.65325	0.76080	1.23763
8SSCL	CSD	70.49628	2.91937	1.14437	0.62070
9SSCL	CSD	134.41689	4.78865	1.16437	0.32280
10SSCL	CSD	135.75337	1.52194	0.37253	0.69051
11SSCL	CSD	123.21106	1.34218	0.28376	0.38219
15SSCL	CSD	128.64255	1.75298	0.56625	2.55907
1kSSCL	control	-	-	-	-
2kSSCL	control	0.43061	0.69659	0.76345	1.51141
3kSSCL	control	0.31557	0.37678	0.49077	1.12930
4kSSCL	control	-	-	-	-
5kSSCL	control	1.34568	0.67931	0.46149	2.86594
6kSSCL	control	0.64749	0.48019	0.73377	1.32627
101SSCL	control	0.14359	0.40999	0.65719	0.92270
102SSCL	control	0.48797	0.28452	0.54387	0.59407
103SSCL	control	0.86836	0.74428	0.91254	0.89348
104SSCL	control	0.69513	0.62861	0.78656	0.83589
