# Supplementary material for: Inflammatory response of leptomeninges to a single cortical spreading depolarization
Source: J Headache Pain. 2024 Jul 16;25(1):113. doi: 10.1186/s10194-024-01823-1 (PMC11251126; doi:10.1186/s10194-024-01823-1)
Supplement: Supplementary file 2 — Supplementary material 2. [file 10194_2024_1823_MOESM2_ESM.docx]

**Supplementary Table S2.** Sequences of primers and annealing temperature used for each primer pair (5′ > 3′).

| Gene | 5'-3' sequence | Temperature, ℃ |
| --- | --- | --- |
| *Ccl2* | Forward - GTCGGCTGGAGAACTACAAGAG  Reverse - GGGTCAAGTTCACATTCAAAGG | 64 |
| *Il1b* | Forward - TCTGTGACTCGTGGGATGAT  Reverse - CACTTGTTGGCTTATGTTCTGTC | 61 |
| *Tnf* | Forward - GTCCAACTCCGGGCTCAGAAT  Reverse - ACTCCCCCGATCCACTCAG | 65 |
| *Cx3cl1* | Forward - ATCACCACCATCACCACCAAC  Reverse - GAGGAACACTTTAAACCCTCACAGA | 61 |
| *Zc3h12a* | Forward - CTCCCTGGAGAGCCAGATGTC  Reverse - GCCAGTACCGATGGCTTGTCTA | 64 |
| *Cnr2* | Forward - GATGGGGCTGCTGAGTGC  Reverse - TATTCCTGGAGTCCACACCGT | 65 |
| *Calca* | Forward - AGTTCTCCCCTTTCCTGGTTGTC  Reverse - CCAGTAGGCGAGCTTCTTCTTCA | 65 |
| *Ywhaz* | Forward - TTGAGCAGAAGACGGAAGGT  Reverse - GAAGCATTGGGGATCAAGAA | 63 |
| *Osbp* | Forward - TCCGGGAGACTTTACCTTCACTT  Reverse - GTGTCACCCTCTTATCAACCACC | 65 |
| *Hprt1* | Forward - CGTCGTGATTAGTGATGATGAAC  Reverse - CAAGTCTTTCAGTCCTGTCCATA | 65 |
