# Supplementary material for: Inflammatory response of leptomeninges to a single cortical spreading depolarization
Source: J Headache Pain. 2024 Jul 16;25(1):113. doi: 10.1186/s10194-024-01823-1 (PMC11251126; doi:10.1186/s10194-024-01823-1)
Supplement: Supplementary file 3 — Supplementary Material 3. [file 10194_2024_1823_MOESM3_ESM.pdf]

Supplementary Table S3. Mean mRNA levels of Ccl2, Zc3h12a, Cx3cl1, Cnr2, Il1b, and Tnf, in the cortical meninges (leptomeninges) of the right (CMR) and left (CML) hemispheres as well as in the parenchyma of the frontal (FCR, FCL), retrosplenial (RCR, RCL) and somatosensory (SSCR, SSCL) cortical regions of the right and left hemispheres in rats CSD and control groups. Significant differences between the groups (Mann-Whitney U test) are marked by dark green (p<0.05) and tendencies (0.05<p<0.1) are marked by light green. For each group, automatically removed outliers and samples removed from processing due to technical problems (ETP) are indicated. The sum of N, outliers and ETP will give the value of the initial group size specified in the methods. Results of comparison with control rats and pairwise interhemispheric comparison are shown.

Comparison with control

Ccl2

	CMR	CML	FCR	FCL	RCR	RCL	SSCR	SSCL
mean_control	0.66440	0.56964	0.47543	0.52613	1.84619	3.13902	0.65202	0.51267
sd_control	0.38164	0.17054	0.24791	0.28082	0.64198	2.77934	0.26838	0.24508
N_control	9	8	9	8	7	8	8	7
outlier_control	5kCMR	2kCML		5kFCL	1kRCR<br>5kRCR	5kRCL	5kSSCR	5kSSCL
ETP_control		104CML	4kFCR	4kFCL	4kRCR	4kRCL	4kSSCR	1kSSCL<br>4kSSCL
mean_CSD	1.09055	31.71464	0.81959	2.80204	2.62712	6.02029	1.13822	91.86596
sd_CSD	0.44232	15.92160	0.57441	1.09381	1.46306	3.00763	1.15493	45.28004
N_CSD	9	11	10	10	9	9	10	11
outlier_CSD			5FCR	8FCL	15RCR	15RCL	5SSCR	
ETP_CSD	6CMR<br>9CMR<br>10CMR	9CML	9FCR	9FCL	2RCR<br>9RCR	8RCL<br>9RCL	9SSCR	9SSCL
MU_p-value	0.03998	0.00003	0.09472	0.00005	0.35105	0.02740	0.76183	0.00006

Zc3h12a

	CMR	CML	FCR	FCL	RCR	RCL	SSCR	SSCL
mean_control	1.13564	1.15071	0.45425	0.54317	1.03052	1.11672	0.51359	0.53753
sd_control	0.30476	0.26230	0.15905	0.11852	0.30938	0.29656	0.08876	0.17149
N_control	8	7	8	7	9	9	8	8
outlier_control	4kCMR<br>101CMR	2kCML<br>4kCML	1kFCR	6kFCL<br>101FCL			2kSSCR	
ETP_control		104CML	4kFCR	4kFCL	4kRCR	4kRCL	4kSSCR	1kSSCL<br>4kSSCL
mean_CSD	1.09616	2.46978	0.47804	0.54195	0.80876	0.65206	0.63114	2.14746
sd_CSD	0.42695	1.37177	0.22253	0.14552	0.54311	0.19476	0.31407	1.16426
N_CSD	9	10	11	11	10	8	11	11
outlier_CSD		6CML				1RCL<br>5RCL		
ETP_CSD	6CMR<br>9CMR<br>10CMR	9CML	9FCR	9FCL	2RCR<br>9RCR	8RCL<br>9RCL	9SSCR	9SSCL
MU_p-value	0.74299	0.03301	0.90389	0.92980	0.31538	0.00790	0.27229	0.00005

Cx3cl1

	CMR	CML	FCR	FCL	RCR	RCL	SSCR	SSCL
mean_control	1.42130	1.17383	0.64629	0.69014	0.93423	1.04252	0.61410	0.66871
sd_control	0.75190	0.62209	0.13572	0.15164	0.20961	0.18320	0.07764	0.15896
N_control	10	8	9	8	9	9	7	8
outlier_control		4kCML		3kFCL			2kSSCR<br>103SSCR	
ETP_control		104CML	4kFCR	4kFCL	4kRCR	4kRCL	4kSSCR	1kSSCL<br>4kSSCL
mean_CSD	0.68773	1.17178	0.56160	0.49096	0.73185	0.78922	0.54312	0.63844
sd_CSD	0.22603	0.57830	0.26061	0.20454	0.33526	0.30365	0.23065	0.26029
N_CSD	8	11	11	11	10	10	11	11
outlier_CSD	8CMR							
ETP_CSD	6CMR<br>9CMR<br>10CMR	9CML	9FCR	9FCL	2RCR<br>9RCR	8RCL<br>9RCL	9SSCR	9SSCL
MU_p-value	0.01166	1.00000	0.29472	0.04088	0.18232	0.07889	0.53601	0.84039

Cnr2

	CMR	CML	FCR	FCL	RCR	RCL	SSCR	SSCL
mean_control	0.67508	0.94122	0.84855	1.02355	0.80818	0.69701	0.78493	1.03044
sd_control	0.24301	0.68093	0.39795	0.51729	0.41807	0.29336	0.31825	0.31285
N_control	8	9	8	9	9	8	8	7
outlier_control	4kCMR<br>5kCMR		1kFCR			5kRCL	5kSSCR	5kSSCL

ETP_control		104CML	4kFCR	4kFCL	4kRCR	4kRCL	4kSSCR	1kSSCL<br>4kSSCL
mean_CSD	0.55540	0.75691	0.91089	1.14628	0.54770	0.70551	1.23200	0.71257
sd_CSD	0.12348	0.31518	0.46977	0.74596	0.24452	0.36323	0.77397	0.29449
N_CSD	7	11	10	11	8	9	10	9
outlier_CSD	5CMR<br>15CMR		5FCR		5RCR<br>6RCR	5RCL	5SSCR	5SSCL<br>15SSCL
ETP_CSD	6CMR<br>9CMR<br>10CMR	9CML	9FCR	9FCL	2RCR<br>9RCR	8RCL<br>9RCL	9SSCR	9SSCL
MU_p-value	0.39689	0.82376	0.82856	0.65563	0.32127	0.74299	0.40825	0.05490

Il1b		
	CMR	CML
mean_control	0.95097	0.91268
sd_control	0.35934	0.28511
N_control	10	9
outlier_control		
ETP_control		104CML
mean_CSD	1.25321	8.32055
sd_CSD	0.21558	3.26096
N_CSD	8	9
outlier_CSD	1CMR	5CML<br>10CML
ETP_CSD	6CMR<br>9CMR<br>10CMR	9CML
MU_p-value	0.08314	0.00004
Tnf		
	CMR	CML
mean_control	0.87477	1.06147
sd_control	0.46242	0.43451
N_control	10	9
outlier_control		
ETP_control		104CML
mean_CSD	1.07885	3.80134
sd_CSD	0.17301	1.87442
N_CSD	8	10
outlier_CSD	15CMR	2CML
ETP_CSD	6CMR<br>9CMR<br>10CMR	9CML
MU_p-value	0.17281	0.00015
Calca		
	CMR	CML
mean_control	2.01901	1.75407
sd_control	1.24373	1.16329
N_control	5	4
outlier_control		
ETP_control	1kCMR<br>5kCMR<br>101CMR<br>103CMR<br>104CMR	1kCML<br>2kCML<br>3kCML<br>6kCML<br>103CML<br>104CML
mean_CSD	1.30417	2.75879
sd_CSD	0.52785	1.53894
N_CSD	3	8
outlier_CSD		
ETP_CSD	2CMR<br>3CMR<br>6CMR<br>7CMR<br>8CMR<br>9CMR<br>10CMR<br>11CMR<br>15CMR	7CML<br>8CML<br>9CML<br>15CML
MU_p-value	0.39286	0.36768

Pairwise comparison

Ccl2

	CMR	CML	FCR	FCL	RCR	RCL	SSCR	SSCL
mean_control	0.83057	0.63646	0.51439	0.84456	3.01624	4.61767	1.48442	0.61680
sd_control	0.70135	0.25619	0.23372	1.10992	2.50015	5.14167	2.35843	0.37178
N_control	9	9	8	8	9	9	8	8
Without_control	104CMR	104CML	1kFCR 4kFCR	1kFCL 4kFCL	4kRCR	4kRCL	1kSSCR 4kSSCR	1kSSCL 4kSSCL
mean_CSD	1.09055	29.58677	1.36092	3.46455	3.92814	7.15545	1.62948	91.86596
sd_CSD	0.44232	15.59047	1.87626	2.42999	3.62791	5.23710	1.96344	45.28004
N_CSD	9	9	11	11	9	9	11	11
Without_CSD	6CMR 9CMR 10CMR	6CML 9CML 10CMR	9FCR	9FCL	2RCR 8RCR 9RCR	2RCL 8RCL 9RCL	9SSCR	9SSCL
W_p-value (control, CSD)*	0.65234	0.00391	0.74219	0.04199	0.49609	0.01953	0.07813	0.00098

Zc3h12a

	CMR	CML	FCR	FCL	RCR	RCL	SSCR	SSCL
mean_control	1.18925	1.20855	0.45425	0.52759	1.03052	1.11672	0.57495	0.53753
sd_control	0.75451	0.69320	0.15905	0.19210	0.30938	0.29656	0.19332	0.17149
N_control	9	9	8	8	9	9	8	8
Without_control	104CMR	104CML	1kFCR 4kFCR	1kFCL 4kFCL	4kRCR	4kRCL	1kSSCR 4kSSCR	1kSSCL 4kSSCL
mean_CSD	1.09616	2.17722	0.47804	0.54195	0.87375	0.91915	0.63114	2.14746
sd_CSD	0.42695	1.07430	0.22253	0.14552	0.53322	0.57032	0.31407	1.16426
N_CSD	9	9	11	11	9	9	11	11
Without_CSD	6CMR 9CMR 10CMR	6CML 9CML 10CMR	9FCR	9FCL	2RCR 8RCR 9RCR	2RCL 8RCL 9RCL	9SSCR	9SSCL
W_p-value (control, CSD)*	0.91016	0.02734	0.38281	0.46484	0.91016	0.73438	0.74219	0.00098

Cx3cl1

	CMR	CML	FCR	FCL	RCR	RCL	SSCR	SSCL
mean_control	1.46978	1.42025	0.63530	0.65663	0.93423	1.04252	0.71391	0.66871
sd_control	0.78076	0.94081	0.14074	0.20193	0.20961	0.18320	0.15434	0.15896
N_control	9	9	8	8	9	9	8	8
Without_control	104CMR	104CML	1kFCR 4kFCR	1kFCL 4kFCL	4kRCR	4kRCL	1kSSCR 4kSSCR	1kSSCL 4kSSCL
mean_CSD	0.76948	1.02634	0.56160	0.49096	0.76265	0.81218	0.54312	0.63844
sd_CSD	0.32380	0.52585	0.26061	0.20454	0.34026	0.31273	0.23065	0.26029
N_CSD	9	9	11	11	9	9	11	11
Without_CSD	6CMR 9CMR 10CMR	6CML 9CML 10CMR	9FCR	9FCL	2RCR 8RCR 9RCR	2RCL 8RCL 9RCL	9SSCR	9SSCL
W_p-value (control, CSD)*	1.00000	0.05469	1.00000	0.36523	0.09766	0.49609	0.38281	0.36523

Cnr2

	CMR	CML	FCR	FCL	RCR	RCL	SSCR	SSCL
mean_control	0.89752	0.94122	0.84855	0.90138	0.80818	0.86127	1.06100	1.25988
sd_control	0.51596	0.68093	0.39795	0.39025	0.41807	0.56402	0.99740	0.71065
N_control	9	9	8	8	9	9	8	8
Without_control	104CMR	104CML	1kFCR 4kFCR	1kFCL 4kFCL	4kRCR	4kRCL	1kSSCR 4kSSCR	1kSSCL 4kSSCL
mean_CSD	0.72124	0.70890	1.18394	1.14628	0.88102	0.93772	1.77524	1.26474
sd_CSD	0.34621	0.32628	1.00934	0.74596	0.67965	0.77109	1.94561	1.36454
N_CSD	9	9	11	11	9	9	11	11
Without_CSD	6CMR 9CMR 10CMR	6CML 9CML 10CMR	9FCR	9FCL	2RCR 8RCR 9RCR	2RCL 8RCL 9RCL	9SSCR	9SSCL
W_p-value (control, CSD)*	0.82031	1.00000	0.74219	0.63770	0.65234	0.57031	0.38281	0.24023

Il1b

	CMR	CML
mean_control	0.92442	0.91268
sd_control	0.37059	0.28511
N_control	9	9
Without_control	104CMR	104CML

mean_CSD	1.33645	11.22180
sd_CSD	0.32097	10.95750
N_CSD	9	9
Without_CSD	6CMR<br>9CMR<br>10CMR	6CML<br>9CML<br>10CMR
W_p-value (control, CSD)*	0.82031	0.00391

Tnf

	CMR	CML
mean_control	0.92828	1.06147
sd_control	0.45645	0.43451
N_control	9	9
Without_control	104CMR	104CML
mean_CSD	1.19219	4.12712
sd_CSD	0.37656	2.74098
N_CSD	9	9
Without_CSD	6CMR<br>9CMR<br>10CMR	6CML<br>9CML<br>10CMR
W_p-value (control, CSD)*	0.09766	0.00391
